# Elevated alanine aminotransferase and low aspartate aminotransferase/alanine aminotransferase ratio are associated with chronic kidney disease among middle-aged women: a cross-sectional study

**DOI:** 10.1186/s12882-020-02144-6

**Published:** 2020-11-10

**Authors:** Hirotaka Ochiai, Takako Shirasawa, Takahiko Yoshimoto, Satsue Nagahama, Akihiro Watanabe, Ken Sakamoto, Akatsuki Kokaze

**Affiliations:** 1 Department of Hygiene, Public Health and Preventive Medicine, Showa University School of Medicine, 1-5-8 Hatanodai, Shinagawa-ku, Tokyo, 142-8555 Japan; 2 All Japan Labor Welfare Foundation, 6-16-11 Hatanodai, Shinagawa-ku, Tokyo, 142-0064 Japan

**Keywords:** Alanine aminotransferase, Aspartate aminotransferase/alanine aminotransferase ratio, Chronic kidney disease

## Abstract

**Background:**

Alanine aminotransferase (ALT) and aspartate aminotransferase (AST) to ALT ratio (AST/ALT ratio) have been shown to be related to non-alcoholic fatty liver disease or insulin resistance, which was associated with chronic kidney disease (CKD). However, it is unclear whether ALT and AST/ALT ratio are associated with CKD. In this study, we examined the relationship of ALT and AST/ALT ratio to CKD among middle-aged females in Japan.

**Methods:**

The present study included 29,133 women aged 40 to 64 years who had an annual health checkup in Japan during April 2013 to March 2014. Venous blood samples were collected to measure ALT, AST, gamma-glutamyltransferase (GGT), and creatinine levels. In accordance with previous studies, ALT > 40 U/L and GGT > 50 U/L were determined as elevated, AST/ALT ratio < 1 was regarded as low, and CKD was defined as estimated glomerular filtration rate < 60 mL/min/1.73 m^2^ and/or proteinuria. Logistic regression model was used to calculate the odds ratio (OR) and 95% confidence interval (CI) for CKD.

**Results:**

“Elevated ALT and elevated GGT” and “elevated ALT and non-elevated GGT” significantly increased the OR for CKD when compared with “non-elevated ALT and non-elevated GGT” (OR: 2.56, 95% CI: 2.10–3.12 and OR: 2.24, 95% CI: 1.81–2.77). Compared with “AST/ALT ratio ≥ 1 and non-elevated GGT”, “AST/ALT ratio < 1 and elevated GGT” and “AST/ALT ratio < 1 and non-elevated GGT” significantly increased the OR for CKD (OR: 2.73, 95% CI: 2.36–3.15 and OR: 1.68, 95% CI: 1.52–1.87). These findings still remained after adjustment for confounders.

**Conclusions:**

Elevated ALT was associated with CKD regardless of GGT elevation. Moreover, low AST/ALT ratio was also associated with CKD independent of GGT elevation.

## Background

The mortality rate in patients with chronic kidney disease (CKD) is reported to be much higher than that in non-CKD patients [1]. In addition, CKD patients have a strong risk of cardiovascular disease [2]. Moreover, health-related quality of life is substantially lower in CKD patients than in the general population [3] and CKD imposes substantial economic burden on affected individuals [4]. Therefore, CKD is a major public health problem.

The prevalence of CKD in Japan was reported to be higher than that in other countries [5]. Imai et al. showed that approximately 13% of Japanese adults were CKD [6]. Moreover, a previous study in Japan demonstrated that the proportion of individuals who developed CKD over 10 years was 19.2% in adults aged 40 years or over [7]. Thus, it is very important to prevent CKD among adults in Japan.

A recent study showed that non-alcoholic fatty liver disease (NAFLD) was associated with CKD [8]. Elevated levels of alanine aminotransferase (ALT), lower aspartate aminotransferase (AST) to ALT ratio (AST/ALT ratio), and elevated gamma-glutamyltransferase (GGT) are commonly used markers of liver injury and non-invasive indicators of NAFLD [9]. Some studies reported that GGT was associated with CKD [10–12]. However, there are no studies reporting on the relationship of elevated ALT and lower AST/ALT ratio to CKD. We hypothesized that elevated ALT and low AST/ALT ratio were associated with CKD and the associations differed by GGT elevation.

Accordingly, the present study investigated the relationship of elevated ALT and low AST/ALT ratio to CKD by considering GGT among adults in Japan.

## Methods

### Subjects

Because the interest of women’s health is increasing worldwide and measures against health problems in middle-aged women are necessary for a healthy life expectancy in old age [13], our study group aimed at preventing lifestyle-related diseases including CKD among middle-aged women. Thus, the present study subjects were women aged 40 to 64 years old who had an annual health checkup performed by the All Japan Labor Welfare Foundation, a health service center in Japan, during April 2013 to March 2014. Of 105,218 subjects, 105,200 agreed with the use of their health checkup data for this study. Of 105,200 participants, serum creatinine levels were measured in 40,658. We excluded 11,525 participants from the analysis due to missing data. Thus, 29,133 participants were analyzed in our study.

Written informed consent for the use of the data in this study was obtained from all participants. The Medical Ethics Committee of Showa University School of Medicine (Approval No. 2133) and the Ethics Committee of the All Japan Labor Welfare Foundation (Approval No. 2-1-0003) approved the study protocol.

### Data collection

A self-administered questionnaire, which was recommended for specific health examination by Ministry of Health, Labour and Welfare, was used to collect the information regarding smoking status and alcohol intake.

Height and weight of each participant were measured to the nearest 0.1 cm and 0.1 kg, respectively. Body mass index (BMI) was calculated as weight (kg) divided by squared height (m). Blood pressure was measured in the sitting position using an automated machine (HEM-907, Omron, Kyoto, Japan).

A venous blood sample was collected from each participant, and stored in a cooler at 4 °C for transportation to an external laboratory (SRL, Tokyo, Japan). High-density lipoprotein cholesterol (HDL-C), low-density lipoprotein cholesterol (LDL-C), triglycerides, blood glucose, hemoglobin A1c (HbA1c), ALT, AST, GGT, and creatinine levels were measured within 24 h of the blood being drawn. HDL-C and LDL-C were determined by a direct method (AU5400, Beckman Coulter, CA, USA), and triglyceride levels were measured by an enzyme method (AU5400, Beckman Coulter). Blood glucose levels were obtained using the hexokinase method (AU5400, Beckman Coulter), and HbA1c was measured by a latex agglutination method (JCA-BM9130, JEOL, Tokyo, Japan). ALT, AST, and GGT were measured by Japan Society of Clinical Chemistry transferable method (AU5400, Beckman Coulter). Serum creatinine levels were determined by an enzyme method (AU5400, Beckman Coulter). The estimated glomerular filtration rate (eGFR) was calculated using the following formula: eGFR = 194 × (serum creatinine^-1.094^) × (age^-0.287^) × 0.739. Urinary analysis was performed by dipstick testing. Urinary protein levels were measured as -, ±, 1+, 2+, or 3 + .

### Definitions

Hypertension was defined as systolic blood pressure ≥ 140 mmHg, diastolic blood pressure ≥ 90 mmHg, or taking medication for hypertension [14]. Dyslipidemia was defined as LDL-C ≥ 140 mg/dl, HDL-C < 40 mg/dl, triglycerides ≥150 mg/dl, or taking medication for dyslipidemia [15]. Diabetes was regarded as having a fasting plasma glucose (≥ 8 h after the last caloric intake [16]) ≥ 126 mg/dl, a random plasma glucose ≥200 mg/dl, an HbA1c (National Glycohemoglobin Standardization Program) ≥ 6.5%, or taking medication for diabetes [15].

In accordance with previous studies, elevated ALT was defined as more than 40 U/L [17, 18]. Low AST/ALT ratio was regarded as less than 1 [19]. Elevated GGT was determined as more than 50 U/L [20, 21]. Based on the criteria of Japan Society for the Study of Obesity [22], obesity was defined as BMI ≥ 25 kg/m^2^. According to previous studies [10, 11, 23], CKD was defined as eGFR < 60 mL/min/1.73 m^2^ and/or urinary protein of 1+ or more (proteinuria). GFR stages G3a-G5 were determined as follows; G3a (eGFR: 45–59), G3b (30–44), G4 (15–29) and G5 (< 15) [24]. Proteinuria stages were classified into the following three stages; A1 (− or ±), A2 (1+ or 2+) and A3 (≥ 3+) [25].

### Statistical analyses

Data were presented as mean (standard deviation) for continuous variables and n (%) for categorical variables. Because the distributions of data for creatinine, ALT, and GGT levels were highly skewed, the data were shown as medians (25 percentile, 75 percentile). Unpaired t-test, Wilcoxon’s rank sum test, or chi-squared test was used to compare characteristics between the group with CKD and the group without CKD. Logistic regression model was used to calculate the odds ratio (OR) and 95% confidence interval (CI) for CKD. First, a crude OR was calculated in the model. Next, we controlled for age, smoking status, alcohol intake, obesity, hypertension, dyslipidemia, and diabetes as confounders [23, 26, 27].

A *P* value < 0.05 was considered statistically significant. Statistical analyses were performed using Statistical Analysis System (SAS) software (version 9.4; SAS Institute Inc., Cary, NC, USA).

## Results

Table 1 shows the characteristics of the study participants. The CKD group (*n* = 2677) was older and was more likely to be obese than the non-CKD group (*n* = 26,456); the mean age and the proportion of obesity were 53.4 years and 29.3% in the CKD group and 50.4 years and 18.5% in the non-CKD group. There were statistically significant differences in smoking status (*P* value < 0.001) and alcohol intake (*P* value < 0.001). The proportions of hypertension, dyslipidemia, and diabetes in the CKD group (36.8, 50.7 and 7.2%) were significantly higher than those in the non-CKD group (28.9, 40.8 and 3.4%). ALT and GGT levels were significantly higher in the CKD group (the median ALT: 18 U/L and the median GGT: 23 U/L) than in the non-CKD group (15 U/L and 18 U/L). In contrast, AST/ALT ratio in the CKD group was significantly lower than that in the non-CKD group (the mean AST/ALT ratio: 1.21 vs. 1.30, *P* value < 0.001).

**Table 1:** Characteristics of study participants

	CKD *(n* = 2677)	Non-CKD (*n* = 26,456)	*P* value^a^
Age (years)	53.4 (6.6)	50.4 (6.9)	< 0.001
Height (cm)	161.6 (8.3)	157.3 (5.9)	< 0.001
Weight (kg)	61.4 (12.4)	54.9 (9.7)	< 0.001
Body mass index (kg/m^2^)	23.4 (4.0)	22.1 (3.7)	< 0.001
Obesity	784 (29.3)	4885 (18.5)	< 0.001
Smoking status			
Current	592 (22.1)	4810 (18.2)	< 0.001
Former	324 (12.1)	1776 (6.7)	
None	1761 (65.8)	19,870 (75.1)	
Alcohol intake			
Everyday	601 (22.5)	4164 (15.7)	< 0.001
Sometimes	778 (29.1)	7932 (30.0)	
None	1298 (48.5)	14,360 (54.3)	
Hypertension	984 (36.8)	7649 (28.9)	< 0.001
Dyslipidemia	1357 (50.7)	10,803 (40.8)	< 0.001
Diabetes	193 (7.2)	888 (3.4)	< 0.001
Creatinine (mg/dl)	0.82 (0.80, 0.90)	0.60 (0.56, 0.69)	< 0.001
eGFR (ml/min/1.73 m^2^)	56.5 (11.4)	81.4 (13.0)	< 0.001
Proteinuria stage			
A1	2302 (86.0)	26,456 (100.0)	< 0.001
A2	334 (12.5)	0 (0.0)	
A3	41 (1.5)	0 (0.0)	
ALT (U/L)	18 (14, 25)	15 (12, 20)	< 0.001
AST/ALT ratio	1.21 (0.39)	1.30 (0.40)	< 0.001
GGT (U/L)	23 (16, 39)	18 (14, 27)	< 0.001

*CKD* Chronic kidney disease, *eGFR* Estimated glomerular filtration rate

*ALT* Alanine aminotransferase, *AST* Aspartate aminotransferase, *GGT* Gamma-glutamyltransferase

Values were mean (standard deviation), median (25th percentile, 75th percentile) or *n* (%)

^a^Unpaired *t* -test, wilcoxon’s rank-sum test or chi-squared test

In the CKD group, proportions of women were 82.0, 6.2, 0.7 and 0.3% for G3a, G3b, G4 and G5. In addition, those of women for A1, A2 and A3 were 86.0, 12.5 and 1.5%.

Table 2 shows the ORs and 95% CIs of elevated ALT, low AST/ALT ratio, and elevated GGT for CKD. Elevated ALT (OR: 2.23, 95% CI: 1.92–2.58) and low AST/ALT ratio (1.82, 1.66–1.99) significantly increased the OR for CKD. Elevated GGT was also significantly associated with CKD (OR: 2.31, 95% CI: 2.06–2.58). These findings persisted even after adjusting for confounding factors (age, smoking status, alcohol intake, obesity, hypertension, dyslipidemia, and diabetes). Even when we adjusted for GGT in addition to the confounding factors, elevated ALT (OR: 1.35, 95% CI: 1.14–1.60) and low AST/ALT ratio (1.37, 1.24–1.52) were significantly associated with CKD.

**Table 2:** Odds ratios and 95% confidence intervals for CKD

	Total N	CKD *n* (%)	Crude	Adjusted
			OR (95% CI)	OR (95% CI)
Elevated ALT				
Yes (40 U/L < ALT)	1307	231 (17.7)	2.23 (1.92–2.58)	1.53 (1.31–1.79)
No (ALT ≤ 40 U/L)	27,826	2446 (8.8)	1.00	1.00
Low AST/ALT ratio				
Yes (AST/ALT ratio < 1)	5533	765 (13.8)	1.82 (1.66–1.99)	1.43 (1.30–1.58)
No (AST/ALT ratio ≥ 1)	23,600	1912 (8.1)	1.00	1.00
Elevated GGT				
Yes (GGT > 50 U/L)	2537	443 (17.5)	2.31 (2.06–2.58)	1.60 (1.42–1.80)
No (GGT ≤ 50 U/L)	26,596	2234 (8.4)	1.00	1.00

*ALT* Alanine aminotransferase, *AST* Aspartate aminotransferase, *GGT* Gamma-glutamyltransferase

*OR* Odds ratio, *CI* Confidence interval, *CKD* Chronic kidney disease

Adjusted for age, smoking status (none, former or current), alcohol intake (none, sometimes or everyday), obesity, hypertension, dyslipidemia, and diabetes

Next, we evaluated the association of the combination of elevated ALT and elevated GGT with CKD (Table 3). “Elevated ALT and elevated GGT” (OR: 2.56, 95% CI: 2.10–3.12) and “non-elevated ALT and elevated GGT” (2.30, 2.02–2.62) significantly increased OR for CKD, compared with “non-elevated ALT and non-elevated GGT”. Significantly increased OR for CKD was also found in “elevated ALT and non-elevated GGT” (OR: 2.24, 95% CI: 1.81–2.77).

**Table 3:** Association of combination of elevated ALT (ALT > 40 U/L) and elevated GGT (GGT > 50 U/L) with CKD

	Total N	CKD *n* (%)	Crude	Adjusted
			OR (95% CI)	OR (95% CI)
ALT > 40 U/L and GGT > 50 U/L	677	126 (18.6)	2.56 (2.10–3.12)	1.70 (1.38–2.10)
ALT > 40 U/L and GGT ≤ 50 U/L	630	105 (16.7)	2.24 (1.81–2.77)	1.57 (1.25–1.96)
ALT ≤ 40 U/L and GGT > 50 U/L	1860	317 (17.0)	2.30 (2.02–2.62)	1.61 (1.41–1.85)
ALT ≤ 40 U/L and GGT ≤ 50 U/L	25,966	2129 (8.2)	1.00	1.00

*ALT* Alanine aminotransferase, *GGT* Gamma-glutamyltransferase, *CKD* Chronic kidney disease

*OR* Odds ratio, *CI* Confidence interval

Adjusted for age, smoking status (none, former or current), alcohol intake (none, sometimes or everyday), obesity, hypertension, dyslipidemia, and diabetes

Table 4 shows the association of the combination of low AST/ALT ratio and elevated GGT with CKD. Compared to “AST/ALT ratio ≥ 1 and non-elevated GGT”, “AST/ALT ratio < 1 and elevated GGT” (OR: 2.73, 95% CI: 2.36–3.15) and “AST/ALT ratio ≥ 1 and elevated GGT” (2.33, 1.98–2.75) were significantly associated with CKD. “AST/ALT ratio < 1 and non-elevated GGT” significantly increased the OR for CKD (OR: 1.68, 95% CI: 1.52–1.87).

**Table 4:** Association of combination of low AST/ALT ratio (AST/ALT ratio < 1) and elevated GGT (GGT > 50 U/L) with CKD

	Total N	CKD *n* (%)	Crude	Adjusted
			OR (95% CI)	OR (95% CI)
AST/ALT ratio < 1 and GGT > 50 U/L	1380	255 (18.5)	2.73 (2.36–3.15)	1.87 (1.60–2.18)
AST/ALT ratio < 1 and GGT ≤ 50 U/L	4153	510 (12.3)	1.68 (1.52–1.87)	1.36 (1.21–1.52)
AST/ALT ratio ≥ 1 and GGT > 50 U/L	1157	188 (16.3)	2.33 (1.98–2.75)	1.57 (1.32–1.86)
AST/ALT ratio ≥ 1 and GGT ≤ 50 U/L	22,443	1724 (7.7)	1.00	1.00

*ALT* Alanine aminotransferase, *AST* Aspartate aminotransferase, *GGT* Gamma-glutamyltransferase

*CKD* Chronic kidney disease, *OR* Odds ratio, *CI* Confidence interval

Adjusted for age, smoking status (none, former or current), alcohol intake (none, sometimes or everyday), obesity, hypertension, dyslipidemia, and diabetes

The results in Tables 3 and 4 remained after adjustment for the confounders. Even when we adjusted for liver disease and heart disease (myocardial infarction and angina), significantly increased ORs for CKD in Tables 3 and 4 persisted.

## Discussion

In the present study, the associations of elevated ALT and low AST/ALT ratio with CKD were examined among women in Japan. As a result, elevated ALT and low AST/ALT ratio were significantly associated with CKD regardless of GGT elevation. This is the first study regarding the relationship of elevated ALT and low AST/ALT ratio to CKD among women in Japan as far as we know. However, these findings should be carefully discussed.

Elevated ALT and low AST/ALT ratio significantly associated with CKD in this study. Alkerwi et al. showed that ALT was significantly higher in participants with CKD than in those without CKD [28], which was consistent with our study result. Elevated ALT and low AST/ALT ratio have been reported to be associated with insulin resistance [17, 19, 29]. A recent study showed that insulin resistance was associated with the occurrence of CKD [30]. These findings support that the present study result was reasonable and elevated ALT.

In our study, elevated GGT was significantly associated with CKD. Previously reported cross-sectional studies showed that GGT elevation significantly increased OR for CKD [12, 31], which was consistent with our study finding. Serum GGT is a biological marker of oxidative stress [32]. Oxidative stress can cause the inflammatory process and accelerate renal injury progression [33]. Thus, elevated GGT might contribute to renal injury, which results in CKD.

Elevated ALT or low AST/ALT ratio was associated with CKD in women with and without elevated GGT, which suggested that elevated ALT or low AST/ALT ratio plays a role in CKD regardless of elevated GGT. The finding suggested that women with elevated ALT or low AST/ALT ratio were more likely to be CKD even when there was no GGT elevation. In addition, the increase of OR for CKD was more pronounced in “low AST/ALT ratio and elevated GGT” women compared to “low AST/ALT ratio and non-elevated GGT” women. The result indicated that the impact of “low AST/ALT ratio and elevated GGT” on CKD was stronger than that of “low AST/ALT ratio and non-elevated GGT” on CKD. In fact, the interaction of low AST/ALT ratio and elevated GGT on CKD was statistically significant. Therefore, it might be effective to consider elevated ALT and low AST/ALT ratio for the early detection of CKD among middle-aged women, especially those with GGT elevation. Because the biological mechanism of the interaction was beyond our study scope, future studies will be required to elucidate the mechanism.

The strength of our study was the large-scale data (approximately 30,000 participants). In contrast, there are some limitations in this study. First, the present study was cross-sectional study, which did not establish the causal relationship of elevated ALT and low AST/ALT ratio with CKD. Second, our study subjects were women in Japan. Thus, the present study findings might not generalize to men or to people of other nationalities. Additionally, we excluded 64,542 women from 105,200 participants in this study because serum creatine levels were not measured, likely due to the fact that the measurement of serum creatine levels was not compulsory in Japan. This could limit the generalizability of our study results to other women. Finally, the information on socioeconomic status such as lower income and lower education [34], serum albumin, creatine kinase and inflammatory markers such as C-reactive protein was not considered in this study. Moreover, there were no imaging data supporting the diagnosis of NAFLD and no data on insulin resistance and oxidative stress. Because these potential confounding factors could affect our study results, it is necessary to consider these factors in future researches.

## Conclusions

Elevated ALT was associated with CKD regardless of GGT elevation. In addition, low AST/ALT ratio was also associated with CKD independent of GGT elevation.

## Data Availability

The datasets used and/or analysed during the current study are available on reasonable request and only after approval by the Ethics Committee of the All Japan Labor Welfare Foundation.

## Abbreviations

CKD: Chronic kidney disease
NAFLD: Non-alcoholic fatty liver disease
ALT: Alanine aminotransferase
AST: Aspartate aminotransferase
AST/ALT ratio: Aspartate aminotransferase to alanine aminotransferase ratio
GGT: Gamma-glutamyltransferase
BMI: Body mass index
HDL-C: High-density lipoprotein cholesterol
LDL-C: Low-density lipoprotein cholesterol
HbA1c: Hemoglobin A1c
eGFR: Estimated glomerular filtration rate
OR: Odds ratio
CI: Confidence interval

## References

[1] Perazella MA, Khan S (2006). Increased mortality in chronic kidney disease: a call to action. Am J Med Sci.

[2] Said S, Hernandez GT (2014). The link between chronic kidney disease and cardiovascular disease. J Nephropathol.

[3] Webster AC, Nagler EV, Morton RL, Masson P (2017). Chronic kidney disease. Lancet..

[4] Jha V, Garcia-Garcia G, Iseki K, Li Z, Naicker S, Plattner B (2013). Chronic kidney disease: global dimension and perspectives. Lancet..

[5] Inaguma D, Imai E, Takeuchi A, Ohashi Y, Watanabe T, Nitta K (2017). Risk factors for CKD progression in Japanese patients: findings from the chronic kidney disease Japan cohort (CKD-JAC) study. Clin Exp Nephrol.

[6] Imai E, Horio M, Watanabe T, Iseki K, Yamagata K, Hara S (2009). Prevalence of chronic kidney disease in the Japanese general population. Clin Exp Nephrol.

[7] Yamagata K, Ishida K, Sairenchi T, Takahashi H, Ohba S, Shiigai T (2007). Risk factors for chronic kidney disease in a community-based population: a 10-year follow-up study. Kidney Int.

[8] Musso G, Gambino R, Tabibian JH, Ekstedt M, Kechagias S, Hamaguchi M (2014). Association of non-alcoholic fatty liver disease with chronic kidney disease: a systematic review and meta-analysis. PLoS Med.

[9] Labayen I, Ruiz JR, Ortega FB, Davis CL, Rodriguez G, Gonzalez-Gross M (2015). Liver enzymes and clustering cardiometabolic risk factors in European adolescents: the HELENA study. Pediatr Obes.

[10] Ryu S, Chang Y, Kim DI, Kim WS, Suh BS (2007). Gamma-Glutamyltransferase as a predictor of chronic kidney disease in nonhypertensive and nondiabetic Korean men. Clin Chem.

[11] Shen ZW, Xing J, Wang QL, Faheem A, Ji X, Li J (2017). Association between serum gamma-glutamyltransferase and chronic kidney disease in urban Han Chinese: a prospective cohort study. Int Urol Nephrol.

[12] Targher G, Kendrick J, Smits G, Chonchol M (2010). Relationship between serum gamma-glutamyltransferase and chronic kidney disease in the United States adult population. Findings from the National Health and nutrition examination survey 2001-2006. Nutr Metab Cardiovasc Dis.

[13] Park S, Yeom HY, Sok SR (2019). Effects of health promoting education program for Korean middle-aged women. J Phys Ther Sci.

[14] Ninomiya T, Kiyohara Y, Kubo M, Tanizaki Y, Doi Y, Okubo K (2005). Chronic kidney disease and cardiovascular disease in a general Japanese population: the Hisayama study. Kidney Int.

[15] Nanri A, Nakagawa T, Kuwahara K, Yamamoto S, Honda T, Okazaki H (2015). Development of risk score for predicting 3-year incidence of type 2 diabetes: Japan epidemiology collaboration on occupational health study. PLoS One.

[16] Kabeya Y, Goto A, Kato M, Matsushita Y, Takahashi Y, Isogawa A (2016). Time spent walking and risk of diabetes in Japanese adults: the Japan public health center-based prospective diabetes study. J Epidemiol.

[17] Chen PH, Chen JD, Lin YC (2009). A better parameter in predicting insulin resistance: obesity plus elevated alanine aminotransferase. World J Gastroenterol.

[18] Chen S, Guo X, Zhang X, Yu S, Yang H, Jiang M (2015). Association between elevated serum alanine aminotransferase and cardiometabolic risk factors in rural Chinese population: a cross-sectional study. BMC Cardiovasc Disord.

[19] Simental-Mendia LE, Rodriguez-Moran M, Gomez-Diaz R, Wacher NH, Rodriguez-Hernandez H, Guerrero-Romero F (2017). Insulin resistance is associated with elevated transaminases and low aspartate aminotransferase/alanine aminotransferase ratio in young adults with normal weight. Eur J Gastroenterol Hepatol.

[20] Iwata T, Arai K, Saito N, Murata K (2013). The association between dietary lifestyles and hepatocellular injury in Japanese workers. Tohoku J Exp Med.

[21] Miyake Y, Eguchi H, Shinchi K, Oda T, Sasazuki S, Kono S (2003). Glucose intolerance and serum aminotransferase activities in Japanese men. J Hepatol.

[22] Examination Committee of Criteria for 'Obesity Disease' in J, Japan Society for the Study of O (2002). New criteria for 'obesity disease’ in Japan. Circ J.

[23] Ohno Y, Ishimura E, Naganuma T, Kondo K, Fukushima W, Mui K (2012). Prevalence of and factors associated with chronic kidney disease (CKD) in Japanese subjects without notable chronic diseases, undergoing an annual health checkup. Kidney Blood Press Res.

[24] Okubo R, Kai H, Kondo M, Saito C, Yoh K, Morito N (2014). Health-related quality of life and prognosis in patients with chronic kidney disease: a 3-year follow-up study. Clin Exp Nephrol.

[25] Ubukata M, Hara M, Nishizawa Y, Fujii T, Nitta K, Ohta A (2018). Prevalence and mortality of chronic kidney disease in lymphoma patients: a large retrospective cohort study. Medicine (Baltimore).

[26] Kazancioglu R (2013). Risk factors for chronic kidney disease: an update. Kidney Int Suppl.

[27] Wakasugi M, Narita I, Iseki K, Moriyama T, Yamagata K, Tsuruya K (2012). Weight gain after 20 years of age is associated with prevalence of chronic kidney disease. Clin Exp Nephrol.

[28] Alkerwi A, Sauvageot N, El Bahi I, Delagardelle C, Beissel J, Noppe S (2017). Prevalence and related risk factors of chronic kidney disease among adults in Luxembourg: evidence from the observation of cardiovascular risk factors (ORISCAV-LUX) study. BMC Nephrol.

[29] Hanley AJ, Wagenknecht LE, Festa A, D'Agostino RB, Haffner SM (2007). Alanine aminotransferase and directly measured insulin sensitivity in a multiethnic cohort: the insulin resistance atherosclerosis study. Diabetes Care.

[30] Ma A, Liu F, Wang C, Liang K, Yan F, Hou X (2018). Both insulin resistance and metabolic syndrome accelerate the progression of chronic kidney disease among Chinese adults: results from a 3-year follow-up study. Int Urol Nephrol.

[31] Chen T, Ren Y, Gao Y, Tian H (2017). Serum gamma-Glutamyl Transferase and ferritin synergistically associated with the rate of chronic kidney disease. Dis Markers.

[32] Fan Y, Jin X, Man C, Gong D (2018). Association of serum gamma-glutamyltransferase with chronic kidney disease risk: a meta-analysis. Free Radic Res.

[33] Modaresi A, Nafar M, Sahraei Z (2015). Oxidative stress in chronic kidney disease. Iran J Kidney Dis.

[34] Zeng X, Liu J, Tao S, Hong HG, Li Y, Fu P (2018). Associations between socioeconomic status and chronic kidney disease: a meta-analysis. J Epidemiol Community Health.

